# Transcriptome analysis of *Polygonum minus* reveals candidate genes involved in important secondary metabolic pathways of phenylpropanoids and flavonoids

**DOI:** 10.7717/peerj.2938

**Published:** 2017-02-28

**Authors:** Kok-Keong Loke, Reyhaneh Rahnamaie-Tajadod, Chean-Chean Yeoh, Hoe-Han Goh, Zeti-Azura Mohamed-Hussein, Zamri Zainal, Ismanizan Ismail, Normah Mohd Noor

**Affiliations:** 1 Institute of Systems Biology, Universiti Kebangsaan Malaysia, Bangi, Malaysia; 2 School of Biosciences and Biotechnology, Faculty of Science and Technology, Universiti Kebangsaan Malaysia, Bangi, Malaysia

**Keywords:** De novo assembly, Hybrid assembly, Illumina sequencing, RNA-seq, *Persicaria minor*, 454 sequencing

## Abstract

**Background:**

*Polygonum minus* is an herbal plant in the Polygonaceae family which is rich in ethnomedicinal plants. The chemical composition and characteristic pungent fragrance of *Polygonum minus* have been extensively studied due to its culinary and medicinal properties. There are only a few transcriptome sequences available for species from this important family of medicinal plants. The limited genetic information from the public expressed sequences tag (EST) library hinders further study on molecular mechanisms underlying secondary metabolite production.

**Methods:**

In this study, we performed a hybrid assembly of 454 and Illumina sequencing reads from *Polygonum minus* root and leaf tissues, respectively, to generate a combined transcriptome library as a reference.

**Results:**

A total of 34.37 million filtered and normalized reads were assembled into 188,735 transcripts with a total length of 136.67 Mbp. We performed a similarity search against all the publicly available genome sequences and found similarity matches for 163,200 (86.5%) of *Polygonum minus* transcripts, largely from *Arabidopsis thaliana* (58.9%). Transcript abundance in the leaf and root tissues were estimated and validated through RT-qPCR of seven selected transcripts involved in the biosynthesis of phenylpropanoids and flavonoids. All the transcripts were annotated against KEGG pathways to profile transcripts related to the biosynthesis of secondary metabolites.

**Discussion:**

This comprehensive transcriptome profile will serve as a useful sequence resource for molecular genetics and evolutionary research on secondary metabolite biosynthesis in Polygonaceae family. Transcriptome assembly of *Polygonum minus* can be accessed at http://prims.researchfrontier.org/index.php/dataset/transcriptome.

## Introduction

Secondary metabolites are organic compounds that are non-vital but indirectly influence plant survival, development and growth. Three major groups of plant secondary metabolites identified by chemical groups are flavonoids and phenolic compounds, terpenoids, and nitrogen/sulfur-containing compounds. Plant secondary metabolites are important natural sources for the development of medicines and natural products. The myriads of plant secondary metabolites reflect the diverse species of plants and their ecological roles, such as adaptation to different environments and defense against biotic stresses (Moore et al., 2014). *Polygonum* is a genus in the Polygonaceae family with up to 300 species, many of which are important as traditional medicinal plants (Narasimhulu, Reddy & Mohamed, 2014).

*Polygonum minus* Huds. (syn. *Persicaria minor*) is a culinary flavoring ingredient common in South East Asia and is also used as a remedy for different maladies ranging from indigestion to poor eyesight (Christapher et al., 2015; George et al., 2014). The leaves of *Polygonum minus* contain high levels of essential oils (72.54%), mainly comprised of aliphatic aldehydes, namely dodecanal (48.18%) and decanal (24.36%) (Yaacob, 1990). Less abundant aldehydes include 1-decanol, 1-dodecanol, undecanal, tetradecanal, 1-undecanol, nonanal, and 1-nonanol (Baharum et al., 2010). Furthermore, the metabolite profiling of *Polygonum minus* leaf revealed many terpenoids and flavonoids with antioxidant activities (Baharum et al., 2010; Goh et al., 2016). The abundance of secondary metabolites in *Polygonum minus* has led to the establishment of hairy root system for the production of plant secondary metabolites (Ashraf et al., 2014). β-caryophyllene was found to be the main sesquiterpenes secreted into the hairy root culture media. These studies showed the potential of developing *Polygonum minus* as a resource to produce natural products.

While many secondary metabolites have been identified in *Polygonum minus*, its biosynthetic pathways remain unclear due to the limited genomic information that is available for the plant. Previously, a total of 3,352 expressed sequence tags (ESTs) were generated from standard cDNA libraries of *Polygonum minus* leaf, root and stem tissues (Roslan et al., 2012). This study indicated the abundance of flavonoid biosynthesis-related genes in the root tissue. The emergence of next generation sequencing has made transcriptomic analysis of plant possible with increasing speed and affordability. RNA-sequencing (RNA-seq) allows novel gene discovery and identification of transcripts of interest in various biological processes. This is especially suitable for many non-model organisms with limited genomic information (Varshney et al., 2009; Ward, Ponnala & Weber, 2012). In the past few years, this platform has been repeatedly utilized to discover and identify genes involved in the biosynthesis of secondary metabolites. For examples, alkaloid biosynthesis in *Uncaria rhynchophylla* (Guo et al., 2014), ginsenoside biosynthesis in *Panax ginseng* (Jayakodi et al., 2015), glucosinolate biosynthesis in *Raphanus sativus* (Wang et al., 2013), biosynthesis of capsaicinoids in *Capsicum frutescens* (Liu et al., 2013), biosynthesis of flavonoids in safflower (Li et al., 2012), and caffeine biosynthesis in *Camellia sinensis* (Shi et al., 2011). To date, there are only two plants from the *Polygonum* genus with RNA-seq data deposited to the public SRA database, namely *Polygonum cuspidatum* (Hao et al., 2012) and *Polygonum tinctorium* (Minami, Sarangi & Thul, 2015). The limited genetic information from this important family of medicinal plants hinders further study on molecular mechanisms underlying the production of bioactive compounds.

To profile transcripts related to the biosynthesis of secondary metabolites in *Polygonum minus*, RNA-seq was performed on the leaf and root tissues. Sequence data generated from 454 and Illumina platforms were assembled, both independently and together, for comparison. This new dataset was compared to all *Polygonum minus* EST transcripts previously deposited to the NCBI database (dated Sep 2014) for validation of the assembly quality. The combined de novo assembly from two different sequencing platforms allowed us to overcome limitations of each technology. We also performed KEGG pathway annotation to identify transcripts related to the biosynthesis of secondary metabolites. This study reveals candidate genes involved in the biosynthesis of secondary metabolites, especially on the biosynthesis of phenylpropanoids and flavonoids in *Polygonum minus* and serves as an invaluable genetic resource for its development as a commercial herbal crop.

## Materials and Methods

### Sample preparation and transcriptome sequencing

Root and leaf tissues of cultivated *Polygonum minus* grown in compost soil without fertilizer were sampled independently from the experimental plot (3°16′14.63″N, 101°41′11.32″E) at Universiti Kebangsaan Malaysia. For the leaf tissue, five expanded young leaves from the apical parts of the plants were collected and pooled as one biological replicate. Samples acquired from 45 day old plants were rinsed with distilled water and flash frozen in liquid nitrogen before stored at −80 °C. Total RNA was isolated using the Lopez-Gomez method with modifications (López-Gómez & Gómez-Lim, 1992) by adding 50% PVP-40 due to high polysaccharide and phenolic compounds in *Polygonum minus*. RNA quality and quantity were assessed using gel electrophoresis, ND-1000 Nanodrop spectrophotometer (Thermo Scientific) and Agilent 2100 Bioanalyzer with a minimum RNA integrity number of 7.

For the root sample, 250 ng of poly(A) RNA was prepared from 800 ng of total RNA using PolyATtract mRNA isolation kit (Promega, Madison, WI, USA) and used as a starting material for the Roche GS FLX sequencing at Malaysia Genome Institute. The cDNA preparation was done according to the cDNA Rapid Library Preparation Method Manual of Roche. The emulsion polymerase chain reaction(PCR) condition was performed using long fragment Lib-A emPCR amplification condition for amplicons that are 550 bp or greater. The conditions are as follows: 94 °C for 4 min, 50 cycles of 94 °C for 30 s and 60 °C for 10 min.

For the leaf sample, total RNA from two biological replicates were used for the Illumina HiSeq™ 2000 sequencing with an average read length of 90 bp through the standard library (200 bp) preparation and paired-end sequencing workflow established at BGI-Shenzhen, China.

### Transcriptome de novo assembly

Raw reads from both 454 pyrosequencing platform and Illumina HiSeq™ 2000 were filtered to remove adapter sequences with sequence pre-processing tools, Cutadapt (Martin, 2011) and Trimmomatic (Bolger, Lohse & Usadel, 2014), respectively. High quality Illumina raw reads with Phred score ≥ 25 were kept for assembly. For root transcriptome, iAssembler pipeline (Zheng et al., 2011), which includes MIRA (Chevreux et al., 2004) and CAP3 (Huang & Madan, 1999) assemblers, was executed with the filtered dataset. The analysis pipeline includes three consecutive runs of MIRA with default parameters followed by CAP3 assembly to obtain the final assembled transcripts. Trinity pipeline (Grabherr et al., 2011) was used to assemble the leaf transcriptome from two leaf libraries, whereas the combined transcriptome was assembled from two leaf libraries and one simulated root library. Assembled reads from the root transcriptome were clipped to 90 bp pseudo reads with 5 bp overlap using an in-house PHP script (http://gitlab.inbiosis.ws/open-source/rnaseq-utils) to simulate Illumina sequencing output for accommodating Trinity assembler short read requirement. For this assembly, leaf raw read datasets were normalized with digital normalization following Khmer 1.0 mRNASeq protocol (Brown et al., 2014).

This project was registered at NCBI’s BioProject with the accession number PRJNA208436. All raw read datasets were deposited to NCBI SRA database (http://www.ncbi.nlm.nih.gov/sra) with the accession number SRX669305 (leaf) and SRX313492 (root) (Loke et al., 2016). Assembled transcripts were deposited to NCBI TSA database (http://www.ncbi.nlm.nih.gov/genbank/tsa) with the accession number GCJZ00000000. The assembled transcripts with annotation can also be accessed at http://prims.researchfrontier.org/index.php/dataset/transcriptome.

### Transcript functional annotation

An annotation pipeline from Trinotate was performed to annotate assembled transcripts (Grabherr et al., 2011). The Trinotate annotation pipeline includes several software packages such as BLASTX, BLASTP, PFAM search, SignalP, and RNAmmer that are essential in transcriptome functional annotation. All analyses were performed in parallel using assembled FASTA sequences.

Functional annotation for all transcripts was performed by running BLASTX similarity search against Trinotate Swiss-Prot protein database (September 2015) with E-value <1e−5 considered as significant hits. For the leaf and combined transcriptomes, Trinotate annotation reports were generated using the standard annotation pipeline (http://trinotate.github.io). Gene Ontology (GO) and Conserved Domain Database (CDD) were used to annotate the transcripts based on similarity. Translated peptides were generated using the Transdecoder program embedded in the Trinity assembly pipeline for protein-based analysis using Eukaryotic Orthologous Group (KOG) classification. All results were deposited into Trinotate-provided SQLite database template and a spreadsheet summary report was generated from Trinotate using BLASTX E-value cutoff of 1e−5.

### KEGG pathway mapping and enrichment analysis

KEGG pathway mapping was performed by associating Enzyme Commission (EC) number from BLAST search results with UniProt database (http://www.uniprot.org/mapping, September, 2015). Metabolite pathway maps with custom color codes were generated using KEGG online Mapper API (http://www.genome.jp/kegg/tool/map_pathway2.html, updated April 1, 2015), with all associated EC number. Pathway enrichment analysis with hypergeometric test was performed by using “Annotate” and “Identify” subprograms in KOBAS version 2.0 with Benjamini–Hochberg correction (Xie et al., 2011). The leaf and root transcriptomes were compared against combined transcriptome as a background for enrichment analysis.

### Transcript abundance estimation

To estimate the relative abundance of transcripts in the leaf and root transcriptomes, filtered 454 (original unclipped) and Illumina raw reads were aligned to the combined transcriptome assembly using RSEM (Li & Dewey, 2011). RSEM statistical model is based on Expectation–Maximization (EM) algorithm to compute maximum likelihood abundance estimates. Transcripts per kilobase million (TPM), which normalizes for transcript length first then sequencing depth, was used as an estimate for the relative expression level (based on proportion of mapped reads) of each transcript in the leaf and root tissues. The same approach was used to identify the list of transcripts present in leaf, root or both tissue types for KEGG pathway mapping by using RSEM estimated count value to determine the presence (TPM > 0) or absence (TPM = 0) of a transcript.

### RT-qPCR of selected transcripts

DNase-treated (Ambion, Huntingdon, UK) RNA (1 μg) was reverse transcribed using iScript™ cDNA Synthesis Kit (Bio-Rad, Hercules, CA, USA) per manufacturer’s protocol. The expression of seven transcripts related to the phenylpropanoid and flavonoid biosynthetic pathways were selected from the transcriptome dataset and specific primer pairs (Table S1) were designed using PrimerBlast software. For RT-qPCR analysis, 1:20 dilution of cDNA was used as template in 20 μL volume and reactions were performed in the iQ™5 Real-Time PCR detection System (Bio-Rad, Hercules, CA, USA) using the iTaq Universal SYBR® Green SuperMix kit (Bio-Rad, Hercules, CA, USA). The amplification was executed with the following cycling program: 3 min at 95 °C, 40 cycles of 10 s at 95 °C, 30 s at 60 °C, and 30 s at 72 °C; and 0.06 s for plate reading at 65 °C followed by a melting curve analysis. Primer efficiencies were determined through standard curves of five cDNA dilution factors in triplicate. *Calcium-Dependent Protein Kinase* (*CDPK*) and *Polyubiquitin* (*UBQ*) were selected as references to normalize expression level. RT-qPCR was performed using three biological replicates, each with three technical replicates. Calculation was based on 2^−ΔΔCt^ method (Nolan, Hands & Bustin, 2006) for fold-change (FC). Correlation plot was generated in MS Excel.

## Results

### Sequencing and assembly

**Figure 1:**
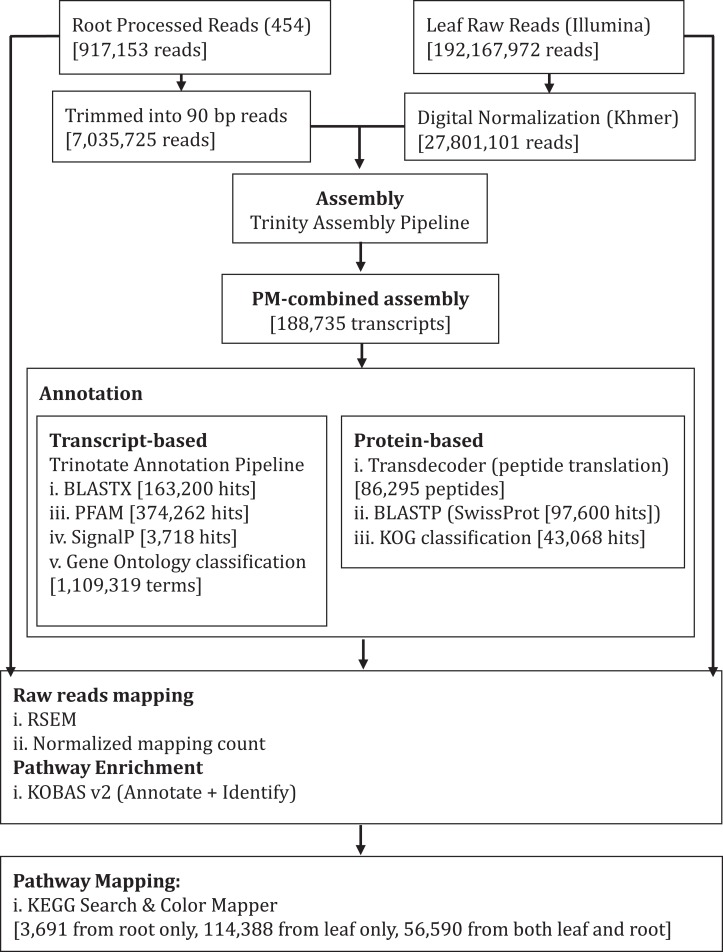
Schematic diagram showing analysis workflow of *Polygonum minus* transcriptome combined assembly and downstream analysis.

**Figure 2:**
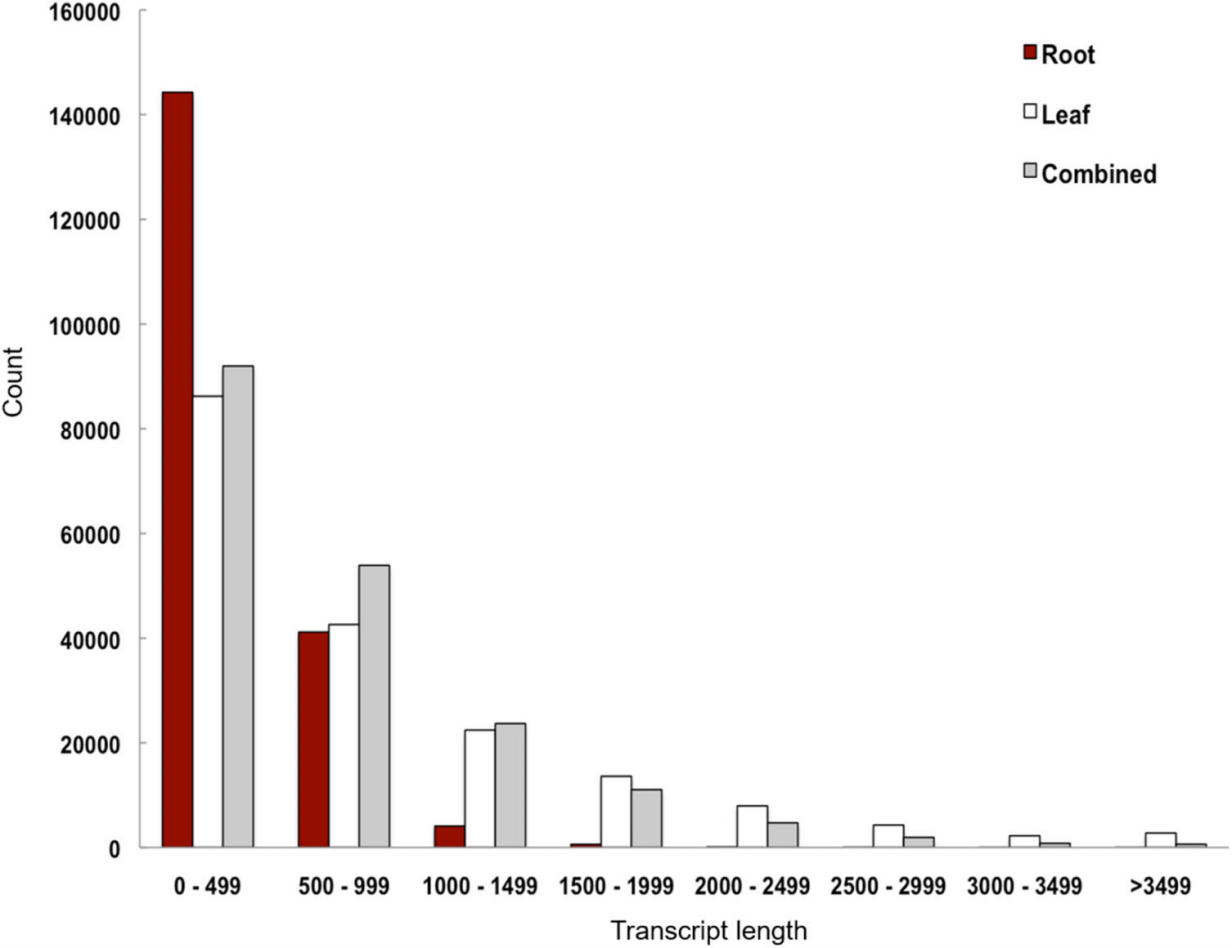
Transcript length distribution of *Polygonum minus* de novo assemblies.

We performed de novo assembly of *Polygonum minus* leaf and root transcriptomes using reads from two different sequencing platforms. Root transcriptome from 454 sequencing showed a typical length distribution ranging from 100 to 600 bp which peaked around 460 bp (Fig. S1). It yielded the lowest coverage with the highest number of redundant reads compared to leaf transcriptome from Illumina sequencing (Table 1). To obtain a more comprehensive reference transcriptome library, we performed combined assembly using normalized Illumina and trimmed 454 reads (Fig. 1). After digital normalization of combined raw reads, 34.37 million high quality reads were assembled into 188,735 transcripts with a median length (N50) of 1,009 bp, and a total length of 136.67 Mbp (Table 1). The number of predicted coding sequence (CDS) also increased for the combined assembly. Combined assembly has a smaller size range compared to leaf assembly alone. This indicates that combined assembly pipeline helps in reducing erroneously long transcripts (>5 kbp) and closing the gaps in the root transcriptome with reduced number of transcript in the combined assembly. This is possibly due to low coverage of root transcriptome resulting in abundant short transcripts (Fig. 2). The combined assembly of the leaf and root transcriptomes also increased the number of transcript, especially for transcripts with length below 1,500 bp. Furthermore, a cross comparison of 3,538 *Polygonum minus* ESTs from a previous study against the combined assembly shows 100% reciprocal hits (Table S2) which supports a comprehensive coverage of the combined assembly.

**Table 1:** Sequence statistics of different *Polygonum minus* transcriptome assemblies.

	Sample (Platform)		Combined assembly
	Root (454)	Leaf (Illumina)	
**Pre-assembly**			
Number of raw reads	1,065,101	192,167,972	48,615,711
Number of processed reads	917,153	191,792,366	34,365,872
Total length (bases)	332,401,206	17,295,117,480	3,061,349,579
Average length (bases)	362	90	90
Median length (N50)	423	90	90
Size range (bases)	100–877	90	90
**Post-assembly**			
Total transcripts	190,269	182,111	188,735
Total length (bases)	85,233,662	157,743,382	136,671,730
Total predicted CDS	45,939	77,010	86,295
Average length (bases)	448	866	724
Median length (N50)	461	1,387	1,009
Size range (bases)	201–3,895	201–17,019	201–12,106

### Similarity search

**Figure 3:**
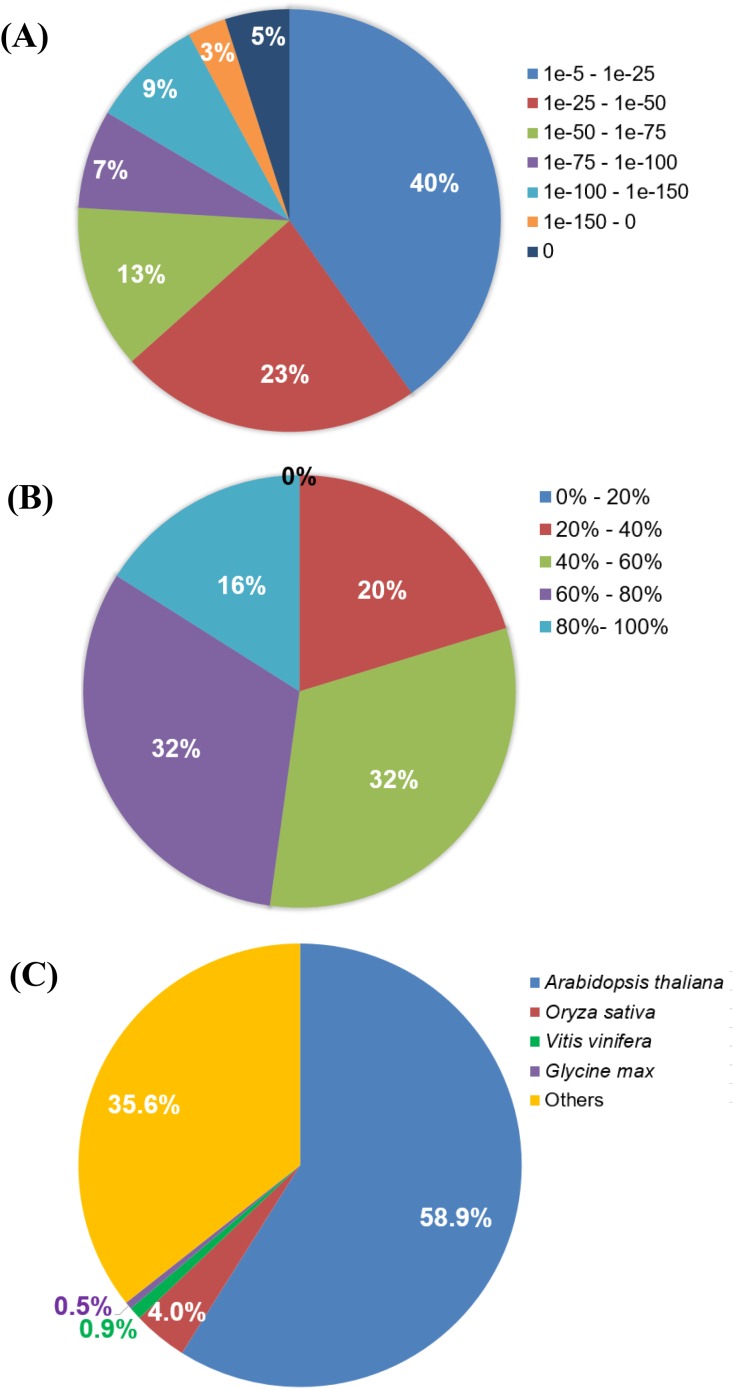
BLASTX analysis of *Polygonum minus* combined assembly. (A) E-value distribution, (B) Similarity distribution, (C) Homologous gene-species distribution.

A summarized assembly and annotation workflow is illustrated in Fig. 1. A total of 163,200 (86.5%) transcripts from the *Polygonum minus* combined (leaf and root) transcriptome assembly were annotated with putative functions based on similarity to sequences in the Swiss-Prot protein database using BLASTX. Among all the BLAST hits, 37% were distributed in the range of E-value lower than 1e−50, 23% with E-value between 1e−25 and 1e−50, and majority of the hits (40%) were distributed in the range of 1e−5–1e−25 (Fig. 3A). BLAST hit similarity distribution analysis showed that majority of the BLAST hits (64%) were in between 40 and 80%; 16% of the transcripts had at least 80% sequence similarity compared to 20% with less than 40% sequence similarity (Fig. 3B). For BLAST hit species distribution (Fig. 3C), we identified that majority of the homologous matches were from *Arabidopsis thaliana* (59%), which is the top most represented plant species in Swiss-Prot database followed by *Oryza sativa* (4%), *Vitis vinifera* (0.9%), and *Glycine max* (0.5%). The rest were classified as “Others” (35.6%) which include mostly hypothetical proteins.

### Functional annotation and classification of the assembled transcripts

**Figure 4:**
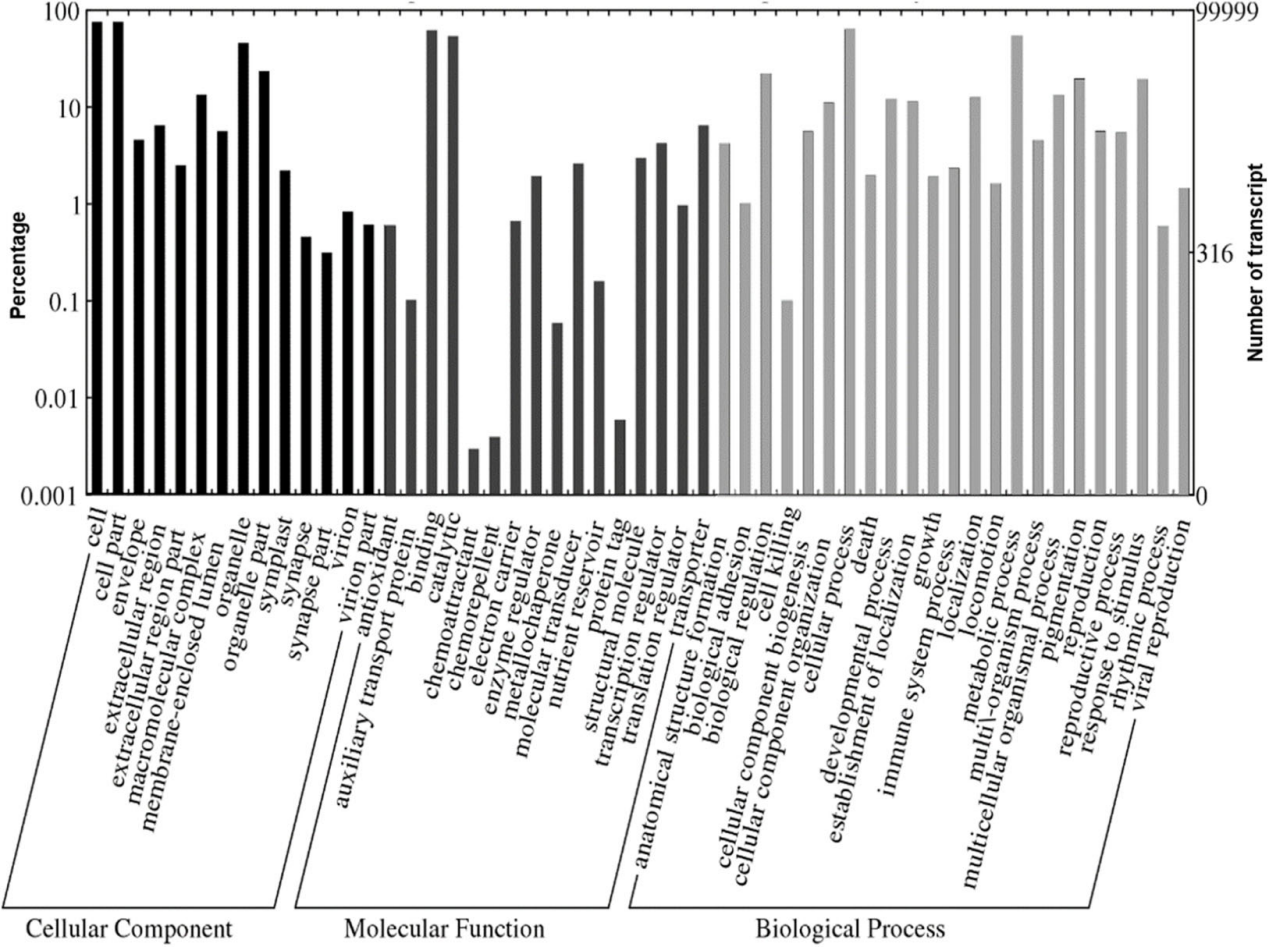
Web Gene Ontology (WEGO) annotation plot of *Polygonum minus* combined assembly.

A total of 1,109,319 GO terms were assigned to the annotated *Polygonum minus* transcripts classified based on BLASTX similarity search. In many cases, multiple terms were assigned to the same transcript, and all of the GO terms were classified into 53 functional groups under three categories of cellular components, biological processes, and molecular functions (Fig. 4). For cellular components, most assignments were cell and cell part (76,052; 76.1%). Within the molecular function category, the GO terms were predominantly assigned to binding (63,382; 63.4%) and catalytic activities (54,855; 54.9%). In biological processes, transcript sequences assigned to cellular (64,870; 64.9%) and metabolic (55,632; 55.6%) processes were the most abundant, followed by biological regulation (22,516; 22.5%), pigmentation (20,141; 20.1%) and response to stimulus (19,847; 19.8%). Similar annotation was observed in KOG classification with the most abundant transcript found in signal transduction mechanism (Fig. S2). These findings revealed that the main functions of the annotated transcripts were responsible for fundamental biological regulation and metabolism common in plants.

### KEGG pathway mapping and gene enrichment analysis

**Figure 5:**
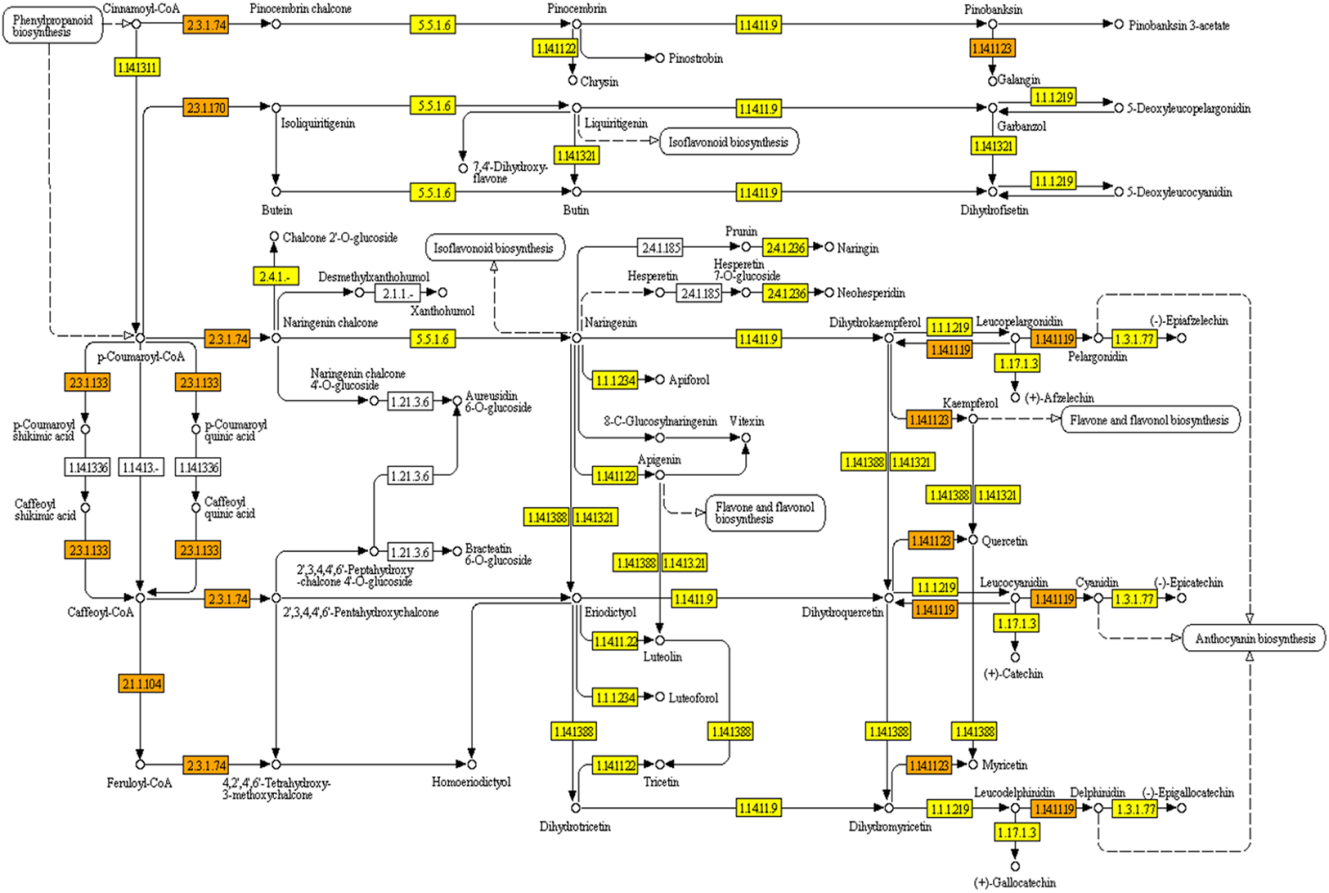
KEGG pathway of flavonoid biosynthesis mapped with combined assembly. Orange/darker shading: found in combined transcriptome and EST library. Yellow/lighter shading: found only in combined transcriptome.

KEGG pathways were mapped with *Polygonum minus* transcripts based on the GO annotation from BLASTX results. A full list of mapped transcripts related to plant secondary metabolite biosynthesis is available as an online material (Data S1). Table 2 shows the KEGG pathways related to selected secondary metabolite biosynthesis which were mapped with *Polygonum minus* combined assembly in comparison with that of publicly available *Polygonum minus* EST sequences. A total of 28,643 transcripts were mapped to 474 out of 949 enzymes in KEGG Orthology (KO) pathways related to the biosynthesis of secondary metabolites. Pathways with majority number of entries mapped include “KO000941 Flavonoid biosynthesis” (17; 89%), “KO00944 Flavone and flavonol biosynthesis” (10; 83%), and “KO00901 Indole alkaloid biosynthesis” (8; 80%). The “KO00940 Phenylpropanoid biosynthesis” pathway has the greatest number of transcript count of 3,753, followed by “KO00906 Carotenoid biosynthesis” (1,259), and “KO00900 Terpenoid backbone biosynthesis” (1,122). The transcript abundance could reflect the importance of these pathways in *Polygonum minus*. Figure 5 depicts the KEGG phenylpropanoid pathway mapped with the combined assembly compared to the previous EST library. This shows that current transcriptome has greatly expanded the number of discovered transcripts related to plant secondary metabolite biosynthesis in *Polygonum minus*.

**Table 2:** KEGG pathways related to selected secondary metabolite biosynthesis mapped with RNA-seq assembly compared to that of publicly available *Polygonum minus* EST sequences.

KEGG Pathway	Total entry	Mapped entry				Transcript count
		EST		RNA-seq		
KO01110 Biosynthesis of secondary metabolites	949	127	13%	474	50%	28,643
KO00909 Sesquiterpenoid and triterpenoid biosynthesis	66	1	2%	12	18%	100
KO00900 Terpenoid backbone biosynthesis	53	11	21%	28	53%	1,122
KO00906 Carotenoid biosynthesis	46	5	11%	13	28%	1,259
KO00904 Diterpenoid biosynthesis	42	0	0%	15	36%	107
KO00940 Phenylpropanoid biosynthesis	32	9	28%	23	72%	3,753
KO00902 Monoterpenoid biosynthesis	22	1	5%	12	55%	54
KO00941 Flavonoid biosynthesis	19	7	37%	17	89%	893
KO00942 Anthocyanin biosynthesis	14	1	7%	4	29%	985
KO00943 Isoflavonoid biosynthesis	13	0	0%	5	38%	34
KO00944 Flavone and flavonol biosynthesis	12	1	8%	10	83%	680

To further examine the relationship between tissue types and biological processes, KEGG pathway enrichment analysis was performed with KOBAS. A list of significant pathways in *Polygonum minus* leaf and root is showed in Table 3. In leaf, pathways related to photosynthesis, primary metabolite metabolism and biosynthesis of secondary metabolites (carotenoids and diterpenoids) were significantly enriched. Conversely, pathways for phenylpropanoid biosynthesis, regulation of actin cytoskeleton, and plant signaling were significantly enriched in the root.

**Table 3:** KEGG metabolic pathway enriched in the leaf and root transcriptomes.

KEGG Pathway	Total entry	Tissue-specific entry	*P*-value
**(A) Leaf transcriptome**			
KO00040—pentose and glucuronate interconversions	305	186	0.00432
KO00196—photosynthesis—antenna proteins	101	68	0.01666
KO00280—valine, leucine, and isoleucine degradation	328	188	0.02246
KO00380—tryptophan metabolism	166	102	0.02422
KO00906—carotenoid biosynthesis	198	118	0.02965
KO00071—fatty acid degradation	377	211	0.03102
KO00500—starch and sucrose metabolism	927	487	0.03692
KO00904—diterpenoid biosynthesis	70	47	0.04188
**(B) Root transcriptome**			
KO00940—phenylpropanoid biosynthesis	645	26	0.00005
KO00360—phenylalanine metabolism	472	20	0.00020
KO04810—regulation of actin cytoskeleton	439	14	0.01727
KO04075—plant hormone signal transduction	1,086	26	0.04266
KO04024—cAMP signaling pathway	411	12	0.04525
KO00562—inositol phosphate metabolism	326	10	0.04919
KO00592—alpha-linolenic acid metabolism	282	9	0.04937

### RT-qPCR validation of RNA-seq relative expression estimation

**Figure 6:**
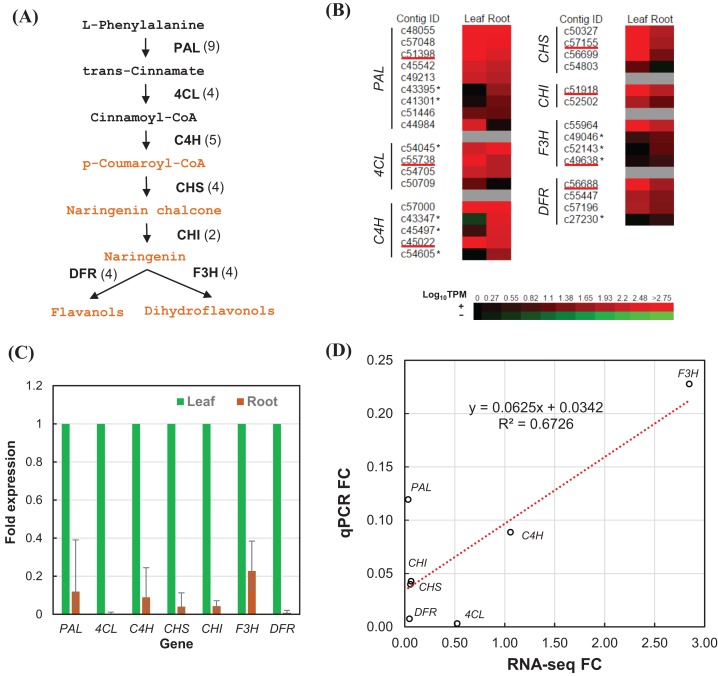
RT-qPCR validation of RNA-seq relative expression estimation. (A) The integrated phenylpropanoid (black) and flavonoid (orange) biosynthesis pathway showing selected genes. Each gene is followed in parentheses by the number of contigs homologous to gene families encoding this enzyme which are expressed in the leaf and root tissues. (B) Expression heatmap of contigs expressed in the leaf and root tissues sorted in descending order of root expression values (Log_10_ TPM). Contigs chosen for RT-qPCR are underlined whereas contigs showing higher expression in the root are marked with an asterisk. (C) RT-qPCR analysis showing the fold of expression level in the root relative to expression level in the leaf. Error bars show the confidence intervals calculated from 1 SE of ΔΔCt. (D) Correlation plot between the RT-qPCR fold-change (FC) compared to FC calculated from TPM values of RSEM estimates. PAL, phenylalanine ammonia-lyase; 4CL, 4-coumarate—CoA ligase; C4H, *trans*-cinnamate-4-monooxygenase; CHS, chalcone synthase; CHI, chalcone isomerase; F3H, flavanone 3-dioxygenase; DFR, dihydroflavonol-4-reductase.

We estimated the expression level of each transcript in the leaf and root tissues based on TPM values (Data S2). To validate the relative expression levels from the transcript abundance estimation, seven transcripts related to phenylpropanoid and flavonoid biosynthetic pathways were chosen for RT-qPCR, namely phenylalanine ammonia-lyase (*PAL*), 4-coumarate–CoA ligase (*4CL*), trans-cinnamate 4-monooxygenase (*C4H*), chalcone synthase (*CHS*), chalcone isomerase (*CHI*), flavanone 3-dioxygenase (*F3H*), and dihydroflavonol-4-reductase (*DFR*) (Fig. 6A). All of the transcripts related to the seven enzymes were identified from the transcriptome sequences based on pairwise sequence alignment BLAT hit result at E-value cutoff of 1e−5 (Table 4, Data S3). Clustered transcripts (contigs) showed variable expression in the leaf and root tissues (Fig. 6B). Most of the contigs had higher TPM values in the leaf tissue; while some contigs were higher in the root tissues, especially for C4H and F3H. Consistently, all of the selected transcripts showed higher expression in the leaf than the root (Fig. 6C). Despite that contig c49638 not showing the expected expression, RT-qPCR results still showed a good correlation (*R*^2^ > 67%) to that of estimated based on RNA-seq TPM values (Fig. 6D).

**Table 4:** Summary of genes involved in phenylpropanoid and flavonoid biosynthesis in *Polygonum minus*.

Gene	KO entry	EC no.	Enzyme name	Number		
				Transcript	Unigene	Contig
PAL	K10775	4.3.1.5	Phenylalanine ammonia-lyase	37	23	13
4CL	K01904	6.2.1.12	4-Coumarate—CoA ligase	15	8	4
C4H	K00487	1.14.13.11	*trans*-Cinnamate-4-monooxygenase	20	10	6
CHS	K00660	2.3.1.74	Chalcone synthase	26	18	12
CHI	K01859	5.5.1.6	Chalcone isomerase	18	3	2
F3H	K00475	1.14.11.9	Flavanone 3-hydroxylase	17	9	6
DFR	K13082	1.1.1.219	Dihydroflavonol 4-reductase	27	13	10

## Discussion

### Hybrid assembly generated a comprehensive reference transcriptome

We performed combined assembly using simulated reads from root transcriptome assembly with Trinity pipeline when the overlap-layout-consensus approach using MIRA failed to combine 454 and Illumina reads. Comparative analyses of de novo transcriptome assemblers based on both simulated and real RNA-seq data (Mundry et al., 2012) suggested that Trinity performed better than MIRA, which is computationally more intensive. Furthermore, MIRA and CAP3 were previously reported to be conservative in merging reads, resulting in a high number of redundant short contigs but fewer chimeric contigs (Clarke et al., 2013). This appeared to be the case for the root transcriptome in which the 190,269 transcripts from iAssembler pipeline were significantly reduced to 26,301 proportionally longer transcripts (Fig. S3) when constructed with Trinity pipeline using the simulated Illumina reads (Table S4). The comparison between the independent and combined assemblies indicates an improvement in the de novo transcriptome quality through the combined assembly approach, based on sequence statistics (Table 1), transcript length distribution (Fig. 1) and validation using EST sequences (Table S2).

The combined assembly provides a more comprehensive reference transcriptome of *Polygonum minus* compared to EST library (Roslan et al., 2012) and cDNA-amplified fragment length polymorphism (cDNA-AFLP) transcript profiling (Ee et al., 2013). All previously found ESTs were covered by the current transcriptome. For example, seven enzymes involved in the flavonoid biosynthesis pathway were identified from previous EST library compared to 17 in current transcriptome study (Table 2). Another two enzymes which were not found in the flavonoid biosynthetic pathway are coumaroylquinate (coumaroylshikimate) 3′-monooxygenase [EC:1.14.13.36] and flavanone 7-*O*-beta-glucosyltransferase [EC:2.4.1.185] (Fig. 5). These two enzymes might be absent or lowly expressed, or no homologous counterpart can be found in the similarity search.

### Pathway enrichment in the leaf and root tissues

Downstream analyses based on combined assembly such as functional annotation and pathway analysis provide insights into the biological processes relevant to the leaf and root tissues. “Photosynthesis” and “Starch and sucrose metabolism” were identified as significantly enriched pathways in leaf (Table 3). The products from these pathways such as xylose can be a substrate for the “Pentose and glucuronate interconversions” pathway. Glucuronate is one of the major sugars required in plant cell wall development (Seitz et al., 2000). These pathways are also involved in the phenolic and antioxidant response pathways and could be related to the high antioxidant and medicinal values of *Polygonum minus* (Mohd Ghazali et al., 2014; Shetty, 2004). Furthermore, the “Carotenoid biosynthesis” and “Diterpenoid biosynthesis” pathways were also enriched in the leaf. This is consistent with the important role of carotenoids and terpenoids in plant protection. Carotenoids can protect the leaf from photo oxidative damage of excessive light by scavenging reactive oxygen species (Cazzonelli, 2011). Terpenes may act as a chemical messenger to regulate the expression of genes in plant defense as well as influencing gene expression of neighboring plants (Cheng et al., 2007). The volatile diterpenes can also protect plants against insects and herbivores (Heiling et al., 2010; Seo et al., 2012). This also supports the discovery of multiple terpenes from chemical analysis of *Polygonum minus* leaf essential oil (Baharum et al., 2010).

“Phenylpropanoid biosynthesis” and “Phenylalanine metabolism” pathways were identified to be enriched in the root transcriptome. Phenylpropanoids are known to be directly involved in plant stress responses towards temperature, drought, UV, and lack of nutrients (Korkina, 2007). The pathway is branched into several important pathways such as flavonoid biosynthetic pathway which plays role in plant resistance (Treutter, 2006). This pathway was highlighted in previous EST analysis which identified 11 ESTs encoding for seven enzymes in the biosynthesis of flavonoids (Fig. 6) (Roslan et al., 2012). Other enriched pathways in the root include “Plant hormone signal transduction” and “cAMP signaling” pathways which indicate the important role of signaling in plant root. Furthermore, inositol phosphate and alpha-linolenic acid related to jasmonic acid are also important in plant signaling and stress response (Witzany, 2006). This supports the importance of root function in detecting environmental signals and transduce them through phytohormones.

### Analysis of transcripts involved in the biosynthesis of phenylpropanoids and flavonoids

Flavonoids are products of the phenylpropanoid metabolism, which bridges between primary and secondary metabolism, through *p*-coumaroyl-CoA that serves as a precursor for flavonoid biosynthesis (Fig. 6A). Based on the abundant transcripts (Data S2) and pathways enrichment analysis in *Polygonum minus* leaf and root tissues (Table 3), we focused on identifying all the transcripts related to the seven important enzymes in phenylpropanoid and flavonoid biosynthesis (Table 4, Data S3). These transcripts can be grouped as unigenes and further clustered into contigs as representative gene families (Grabherr et al., 2011). In general, most of the contigs related to phenylpropanoid and flavonoid biosynthesis were more abundant in the leaf tissue based on the normalized TPM values (Fig. 6B).

However, certain contigs did show otherwise which indicate differential regulation of different gene families in the leaf and root tissues as proposed in previous study (Roslan et al., 2012). The EST study by Roslan et al. (2012) showed higher expression of three genes related to flavonoid biosynthesis in *Polygonum minus* root, namely *CHS*, flavonol synthase (*FLS*), and leucoanthocyanidin dioxygenase (*LDOX*). Indeed, our transcriptome analysis also showed higher expression of certain transcripts in the root tissue (Data S2 and S3). The comprehensive transcriptome analysis and robust correlation with RT-qPCR results in current study showed that the expression of transcripts related to the biosynthesis of phenylpropanoids and flavonoids in *Polygonum minus* were more prevalent in the leaf than the root tissue. This finding is consistent with recent metabolite profiling study in different tissues of *Polygonum minus* which showed that the phenolic content and antioxidant activity in the leaf extract is much higher than that of root extract (Ahmad et al., 2014).

The lack of a reference genome for *Polygonum minus* has made it difficult to determine the exact number of genes involved in the metabolic pathways. Here, a large number of candidate transcripts could be matched with known enzymes in public databases which provides a substantial gene resource for further research in *Polygonum minus*. Our study also provides reference sequences for evolutionary analyses of metabolomes among the members of Polygonaceae family which is rich in ethnomedicinal plants.

## Concluding Remarks

In this study, we described the first comprehensive transcriptome profile of *Polygonum minus* leaf and root tissues for the curation of secondary metabolite-related transcripts (Data S1). We compared the annotation results of three *Polygonum minus* assemblies (leaf, root, and combined) with that of previous EST library. This comparison provides a useful resource for gene discovery using *Polygonum minus* combined assembly. We have established a reference transcriptome profile of *Polygonum minus* with annotations of transcript descriptions for future investigation on specific processes or pathways, especially on the effect of environmental stresses and the biosynthesis of secondary metabolites. We also identified some of the enriched metabolite pathways in the root and leaf tissues. The identification of transcripts related to secondary metabolite biosynthesis will aid in further exploitation of the genetic resource from this herbal plant for future biotechnological development.

## Supplemental Information

10.7717/peerj.2938/supp-1 Supplemental Information 1 Supplementary Figures.

10.7717/peerj.2938/supp-2 Supplemental Information 2 Supplementary Tables.

10.7717/peerj.2938/supp-3 Supplemental Information 3 Data S1. KEGG pathway mapping results.

10.7717/peerj.2938/supp-4 Supplemental Information 4 Data S2. RSEM estimation of transcript abundance in the leaf and root tissues with functional annotation.

10.7717/peerj.2938/supp-5 Supplemental Information 5 Data S3. Profiling of candidate genes related to the biosynthesis of phenylpropanoids and flavonoids.

## Additional Information and Declarations

### DNA Deposition

The following information was supplied regarding the deposition of DNA sequences: BioProject: PRJNA208436. BioSample: SAMN02211834. Sequence Read Archive: SRR917962.

### Data Deposition

The following information was supplied regarding data availability: Assembled transcripts can be found at http://prims.researchfrontier.org/index.php/dataset/transcriptome.

## References

[ref-1] Ahmad R, Baharum SN, Bunawan H, Lee M, Noor N, Rohani ER, Ilias N, Zin N (2014). Volatile profiling of aromatic traditional medicinal plant, *Polygonum minus* in different tissues and its biological activities. Molecules.

[ref-2] Ashraf MF, Che Mohd Zain CR, Zainal Z, Mohd Noor N, Anuar N, Markom M, Ismail I (2014). Establishment of *Persicaria minor* hairy roots and analysis of secreted β-caryophyllene in medium broth. Plant Cell, Tissue and Organ Culture.

[ref-3] Baharum SN, Bunawan H, Ghani MA, Mustapha WAW, Noor NM (2010). Analysis of the chemical composition of the essential oil of *Polygonum minus* Huds. Using two-dimensional gas chromatography-time-of-flight mass spectrometry (GC-TOF MS). Molecules.

[ref-4] Bolger AM, Lohse M, Usadel B (2014). Trimmomatic: a flexible trimmer for Illumina sequence data. Bioinformatics.

[ref-5] Brown CT, Crusoe MR, Edvenson G, Fish J, Howe A, McDonald E, Nahum J, Nanlohy K, Ortiz-Zuazaga H, Pell J, Simpson J, Scott C, Srinivasan RR, Zhang Q (2014). The khmer software package: enabling efficient sequence analysis. figshare.

[ref-6] Cazzonelli CI (2011). Carotenoids in nature: insights from plants and beyond. Functional Plant Biology.

[ref-7] Cheng A-X, Lou Y-G, Mao Y-B, Lu S, Wang L-J, Chen X-Y (2007). Plant terpenoids: biosynthesis and ecological functions. Journal of Integrative Plant Biology.

[ref-8] Chevreux B, Pfisterer T, Drescher B, Driesel AJ, Müller WEG, Wetter T, Suhai S (2004). Using the miraEST assembler for reliable and automated mRNA transcript assembly and SNP detection in sequenced ESTs. Genome Research.

[ref-9] Christapher P, Parasuraman S, Christina JMA, Asmawi MZ, Vikneswaran M (2015). Review on *Polygonum minus* Huds, a commonly used food additive in Southeast Asia. Pharmacognosy Research.

[ref-10] Clarke K, Yang Y, Marsh R, Xie LL, Zhang KK (2013). Comparative analysis of de novo transcriptome assembly. Science China Life Sciences.

[ref-11] Ee S-F, Oh J-M, Mohd Noor N, Kwon T-R, Mohamed-Hussein Z-A, Ismail I, Zainal Z (2013). Transcriptome profiling of genes induced by salicylic acid and methyl jasmonate in *Polygonum minus*. Molecular Biology Reports.

[ref-12] George A, Ng CP, O’Callaghan M, Jensen GS, Wong HJ (2014). In vitro and ex-vivo cellular antioxidant protection and cognitive enhancing effects of an extract of *Polygonum minus* Huds (LineminusTM) demonstrated in a Barnes Maze animal model for memory and learning. BMC Complementary and Alternative Medicine.

[ref-13] Goh H-H, Khairudin K, Sukiran NA, Normah MN, Baharum SN (2016). Metabolite profiling reveals temperature effects on the VOCs and flavonoids of different plant populations. Plant Biology.

[ref-14] Grabherr MG, Haas BJ, Yassour M, Levin JZ, Thompson DA, Amit I, Adiconis X, Fan L, Raychowdhury R, Zeng Q, Chen Z, Mauceli E, Hacohen N, Gnirke A, Rhind N, di Palma F, Birren BW, Nusbaum C, Lindblad-Toh K, Friedman N, Regev A (2011). Full-length transcriptome assembly from RNA-Seq data without a reference genome. Nature Biotechnology.

[ref-15] Guo Q, Ma X, Wei S, Qiu D, Wilson IW, Wu P, Tang Q, Liu L, Dong S, Zu W (2014). De novo transcriptome sequencing and digital gene expression analysis predict biosynthetic pathway of rhynchophylline and isorhynchophylline from *Uncaria rhynchophylla*, a non-model plant with potent anti-alzheimer’s properties. BMC Genomics.

[ref-16] Hao D, Ma P, Mu J, Chen S, Xiao P, Peng Y, Huo L, Xu L, Sun C (2012). De novo characterization of the root transcriptome of a traditional Chinese medicinal plant *Polygonum cuspidatum*. Science China Life Sciences.

[ref-17] Heiling S, Schuman MC, Schoettner M, Mukerjee P, Berger B, Schneider B, Jassbi AR, Baldwin IT (2010). Jasmonate and ppHsystemin regulate key malonylation steps in the biosynthesis of 17-hydroxygeranyllinalool diterpene glycosides, an abundant and effective direct defense against herbivores in *Nicotiana attenuata*. Plant Cell.

[ref-18] Huang X, Madan A (1999). CAP3: a DNA sequence assembly program. Genome Research.

[ref-19] Jayakodi M, Lee S-C, Lee YS, Park H-S, Kim N-H, Jang W, Lee HO, Joh HJ, Yang T-J (2015). Comprehensive analysis of *Panax ginseng* root transcriptomes. BMC Plant Biology.

[ref-20] Korkina LG (2007). Phenylpropanoids as naturally occurring antioxidants: from plant defense to human health. Cellular and Molecular Biology.

[ref-21] Li B, Dewey CN (2011). RSEM: accurate transcript quantification from RNA-Seq data with or without a reference genome. BMC Bioinformatics.

[ref-22] Li H, Dong Y, Yang J, Liu X, Wang Y, Yao N, Guan L, Wang N, Wu J, Li X (2012). De novo transcriptome of safflower and the identification of putative genes for oleosin and the biosynthesis of flavonoids. PLoS ONE.

[ref-23] Liu S, Li W, Wu Y, Chen C, Lei J (2013). De novo transcriptome assembly in chili pepper (*Capsicum frutescens*) to identify genes involved in the biosynthesis of capsaicinoids. PLoS ONE.

[ref-24] Loke K-K, Rahnamaie-Tajadod R, Yeoh C-C, Goh H-H, Mohamed-Hussein ZA, Mohd Noor N, Zainal Z, Ismail I (2016). RNA-seq analysis for secondary metabolite pathway gene discovery in *Polygonum minus*. Genomics Data.

[ref-25] López-Gómez R, Gómez-Lim MA (1992). A method for extracting intact RNA from fruits rich in polysaccharides using ripe mango mesocarp. HortScience.

[ref-26] Martin M (2011). Cutadapt removes adapter sequences from high-throughput sequencing reads. EMBnet.journal.

[ref-27] Minami Y, Sarangi BK, Thul ST (2015). Transcriptome analysis for identification of indigo biosynthesis pathway genes in *Polygonum tinctorium*. Biologia (Poland).

[ref-28] Mohd Ghazali MA, Al-Naqeb G, Krishnan Selvarajan K, Hazizul Hasan M, Adam A (2014). Apoptosis induction by *Polygonum minus* is related to antioxidant capacity, alterations in expression of apoptotic-related genes, and S-phase cell cycle arrest in HepG2 cell line. BioMed Research International.

[ref-29] Moore BD, Andrew RL, Külheim C, Foley WJ (2014). Explaining intraspecific diversity in plant secondary metabolites in an ecological context. New Phytologist.

[ref-30] Mundry M, Bornberg-Bauer E, Sammeth M, Feulner PGD (2012). Evaluating characteristics of de novo assembly software on 454 transcriptome data: a simulation approach. PLoS ONE.

[ref-31] Narasimhulu G, Reddy KK, Mohamed J (2014). The genus Polygonum (Polygonaceae): An ethnopharmacological and phytochemical perspectives: review. International Journal of Pharmacy and Pharmaceutical Sciences.

[ref-32] Nolan T, Hands RE, Bustin SA (2006). Quantification of mRNA using real-time RT-PCR. Nature Protocols.

[ref-33] Roslan ND, Yusop JM, Baharum SN, Othman R, Mohamed-Hussein Z-A, Ismail I, Noor NM, Zainal Z (2012). Flavonoid biosynthesis genes putatively identified in the aromatic plant *Polygonum minus* via expressed sequences tag (EST) analysis. International Journal of Molecular Sciences.

[ref-34] Seitz B, Klos C, Wurm M, Tenhaken R (2000). Matrix polysaccharide precursors in Arabidopsis cell walls are synthesized by alternate pathways with organ-specific expression patterns. Plant Journal.

[ref-35] Seo S, Gomi K, Kaku H, Abe H, Seto H, Nakatsu S, Neya M, Kobayashi M, Nakaho K, Ichinose Y, Mitsuhara I, Ohashi Y (2012). Identification of natural diterpenes that inhibit bacterial wilt disease in tobacco, tomato and arabidopsis. Plant and Cell Physiology.

[ref-36] Shetty K (2004). Role of proline-linked pentose phosphate pathway in biosynthesis of plant phenolics for functional food and environmental applications: a review. Process Biochemistry.

[ref-37] Shi CY, Yang H, Wei CL, Yu O, Zhang ZZ, Jiang CJ, Sun J, Li YY, Chen Q, Xia T, Wan XC (2011). Deep sequencing of the Camellia sinensis transcriptome revealed candidate genes for major metabolic pathways of tea-specific compounds. BMC Genomics.

[ref-38] Treutter D (2006). Significance of flavonoids in plant resistance: a review. Environmental Chemistry Letters.

[ref-39] Varshney RK, Nayak SN, May GD, Jackson SA (2009). Next-generation sequencing technologies and their implications for crop genetics and breeding. Trends in Biotechnology.

[ref-40] Wang Y, Pan Y, Liu Z, Zhu X, Zhai L, Xu L, Yu R, Gong Y, Liu L (2013). De novo transcriptome sequencing of radish (*Raphanus sativus* L.) and analysis of major genes involved in glucosinolate metabolism. BMC Genomics.

[ref-41] Ward JA, Ponnala L, Weber CA (2012). Strategies for transcriptome analysis in nonmodel plants. American Journal of Botany.

[ref-42] Witzany G (2006). Plant communication from biosemiotic perspective: differences in abiotic and biotic signal perception determine content arrangement of response behavior. Context determines meaning of meta-, inter- and intraorganismic plant signaling. Plant Signaling and Behavior.

[ref-43] Xie C, Mao X, Huang J, Ding Y, Wu J, Dong S, Kong L, Gao G, Li C-Y, Wei L (2011). KOBAS 2.0: a web server for annotation and identification of enriched pathways and diseases. Nucleic Acids Research.

[ref-44] Yaacob KB (1990). Essential oil of *Polygonum minus* Huds. Journal of Essential Oil Research.

[ref-45] Zheng Y, Zhao L, Gao J, Fei Z (2011). iAssembler: a package for de novo assembly of Roche-454/Sanger transcriptome sequences. BMC Bioinformatics.

