# Supplementary material for: Transcriptome analysis of Polygonum minus reveals candidate genes involved in important secondary metabolic pathways of phenylpropanoids and flavonoids
Source: PeerJ. 2017 Feb 28;5:e2938. doi: 10.7717/peerj.2938 (PMC5333554; doi:10.7717/peerj.2938)
Supplement: Supplemental Information 1 [file peerj-05-2938-s001.pdf]

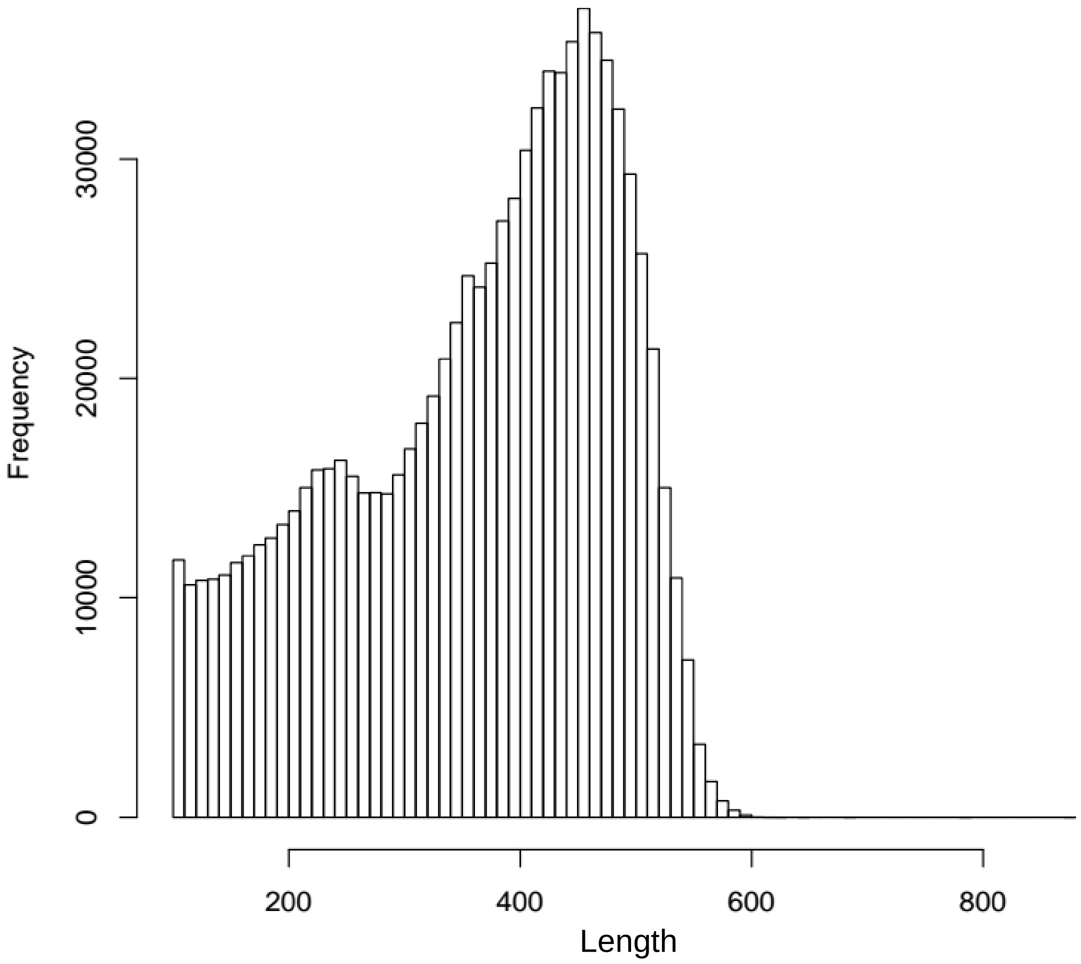

Figure S1. Length distribution of 454 raw reads

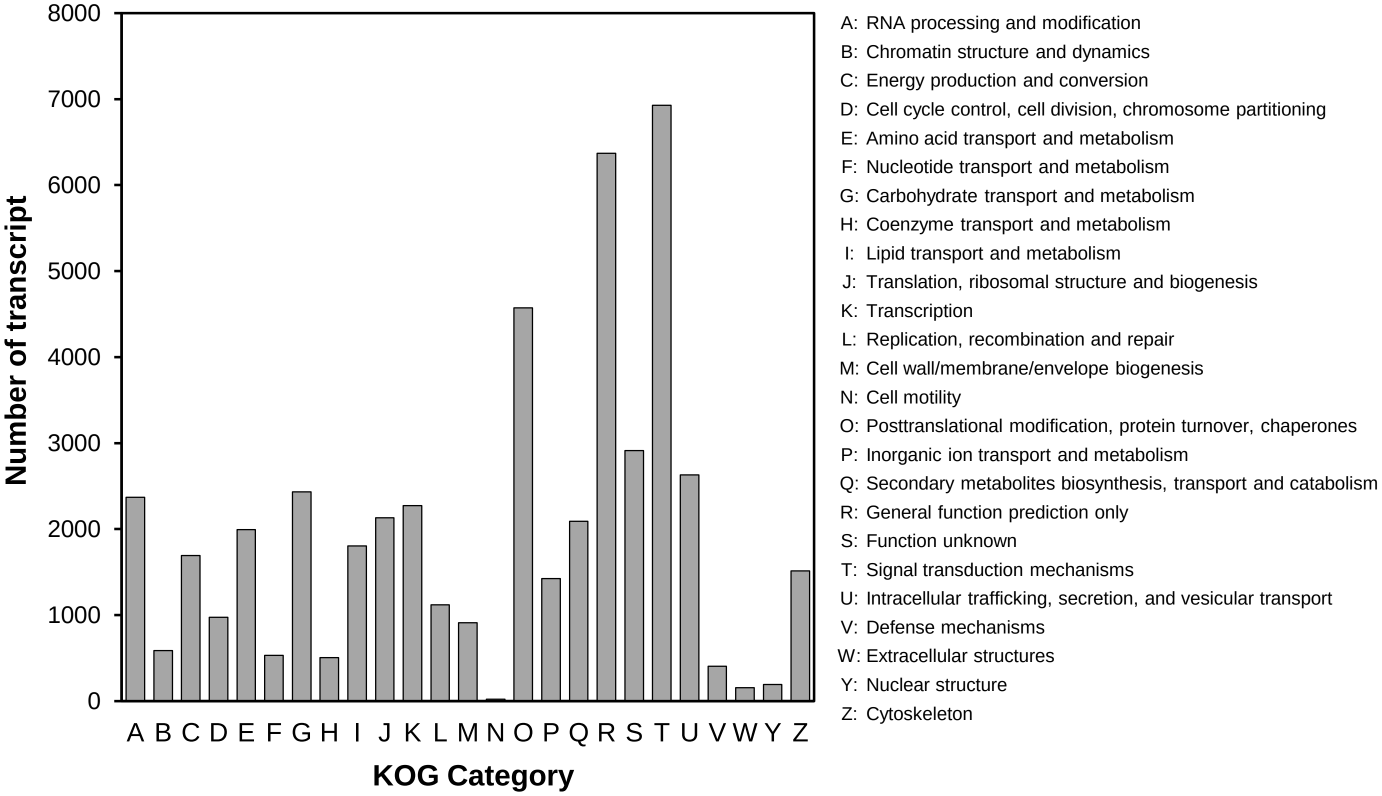

Figure S2. Eukaryotic Orthologous Groups (KOG) classification with translated peptides from *P. minus* combined assembly.

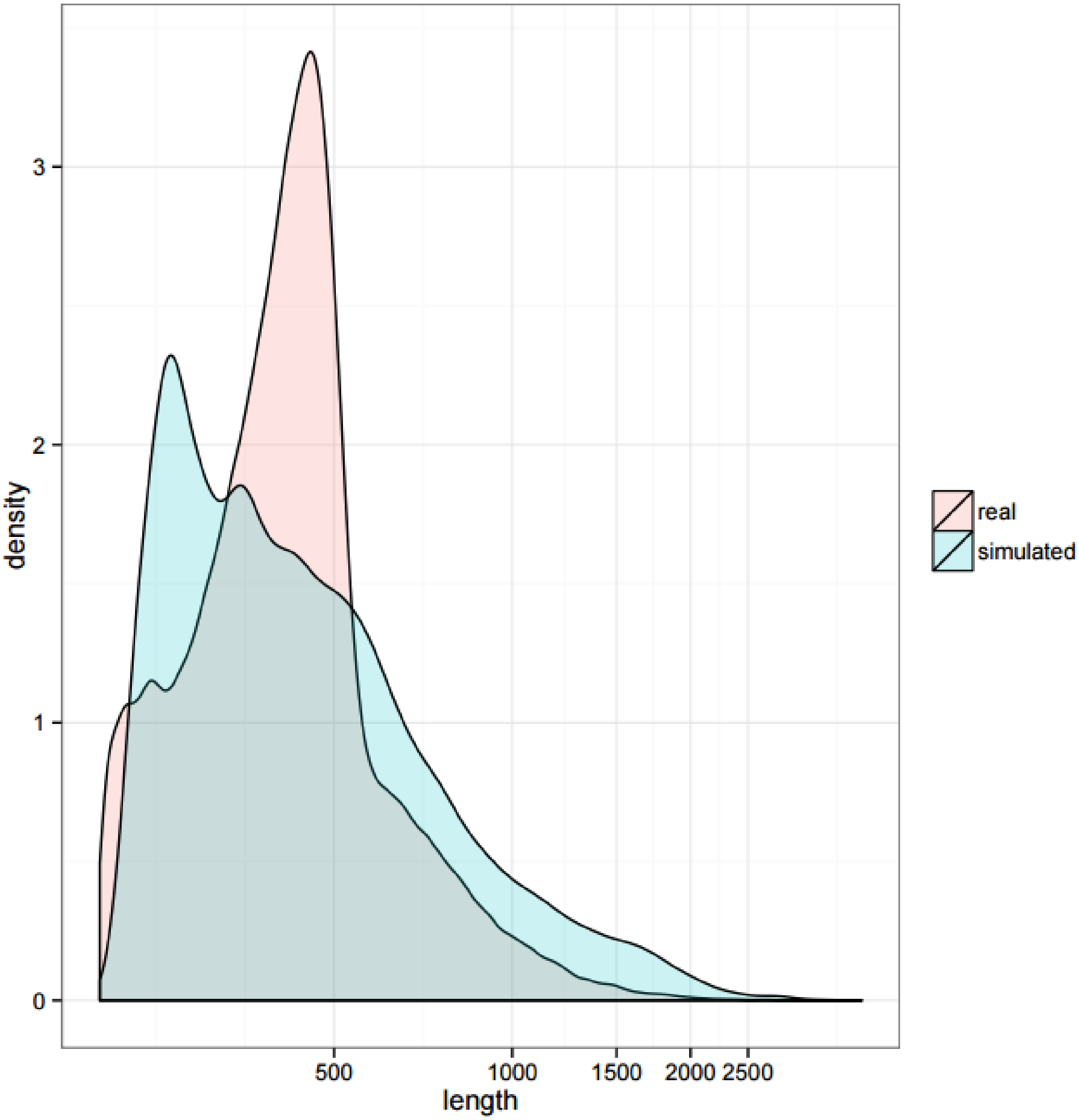

Figure S3. Length distribution of transcripts assembled using actual 454 reads through iAssembler pipeline (real) compared to simulated Illumina reads from assembled root transcriptome through Trinity pipeline
