# Supplementary material for: Transcriptome analysis of Polygonum minus reveals candidate genes involved in important secondary metabolic pathways of phenylpropanoids and flavonoids
Source: PeerJ. 2017 Feb 28;5:e2938. doi: 10.7717/peerj.2938 (PMC5333554; doi:10.7717/peerj.2938)
Supplement: Supplemental Information 2 [file peerj-05-2938-s002.pdf]

Supplementary Table S1 Primer sequences used in the qRT-PCR

Transcript id	Gene	Symbol	Primer	Sequence (5'→ 3')
c51398_g3_i1	Phenylalanine ammonia-lyase	PAL	Forward	TTGCAGCCCCAACTACCTGC
			Reverse	CTTCCTCCACCGCCTTTGGG
c55738_g3_i1	4-Coumarate--CoA ligase	4CL	Forward	CTTGTGGACGAGCAGGACGG
			Reverse	AGCCTGATAACGAGCGTGGC
c45022_g1_i1	Trans-cinnamate 4-monooxygenase	C4H	Forward	CTATCACCGCCGTTCCGGTC
			Reverse	ACAAGAGGCGCAGAGGAGGA
c57155_g2	Chalcone synthase	CHS	Forward	GCGCTCGACATGTTCCCGTA
			Reverse	CAAGCTGGGGCTCAAGGAGG
c51918_g3	Chalcone isomerase	CHI	Forward	ACACCAAGCCACTTCCTCGAC
			Reverse	TGGCGGTGTGCTTGATTTGC
c49638_g1_i4	Flavanone 3-hydroxylase	F3H	Forward	TCCACCATGGAAACCCACCC
			Reverse	CGGGTGACTCGGAAATGGGA
c56688_g2_i1	Dihydroflavonol-4-reductase	DFR	Forward	AAGTGGGTGTTCGCCGTAGG
			Reverse	CCGTCCGTGACCCTACCAAC
c50203_g2_i1	Polyubiquitin 11	UBQ11	Forward	GCGTGGTGGGATGCAGATTTTCG
			Reverse	CTGAATCTTGGCCTTCACATTGTC
c56675_g6_i3-7	Calcium-dependent protein kinase	CDPK	Forward	GCCTTTGCTGCTATGTGGGC
			Reverse	AACGGCTGATGACTTCGCCA

Supplementary Table S2 Comparison between *P. minus* EST dataset with combined assembly

	EST (NCBI)*	Combined assembly
Number of unique transcripts	3,538	188,735
Total length (bases)	2,060,435	136,671,730
Average length (bases)	619	724
N50	582	1,009
Size range (bases)	201-3895	201-12,106

**P. minus* EST sequences were downloaded from NCBI as of 05 Sept 2014.

*All EST has significant homologues with unique transcripts (BLASTN, E value 1e-06).

1,722 unique transcripts are best reciprocal BLASTX hits (100% similarity) with 1,982 ESTs.

Supplementary Table S3 Sequence statistics of root 454 iAssembler assembly compared with Trinity assembly from simulated root 454 reads

Assembly	Real (454)	Simulated (Illumina)
**Pre-assembly**		
Number of raw reads	1,065,101	-
Number of processed reads	917,153	5,408,409
Total length (bases)	332,401,206	446,803,875
Average length (bases)	362	90
Median length (N50)	423	90
Size range (bases)	100-877	90
**Post-assembly**		
Total transcripts	190,269	26,301
Total length (bases)	85,233,662	13,503,569
Average length (bases)	448	513
Median length (N50)	461	567
Size range (bases)	201-3,895	224-3,413
